# Patient communication and experiences in cancer clinical drug trials: a mixed-method study at a specialist clinical trials unit

**DOI:** 10.1186/s13063-023-07284-2

**Published:** 2023-06-13

**Authors:** Rowan Forbes Shepherd, Ashleigh Bradford, Marian Lieschke, Kylie Shackleton, Amelia Hyatt

**Affiliations:** 1 Department of Health Services Research and Implementation Science, Peter MacCallum Cancer Centre, 305 Grattan Street, Melbourne, VIC 3000 Australia; 2 Parkville Cancer Clinical Trials Unit, Peter MacCallum Cancer Centre, Melbourne, VIC 3000 Australia; 3 Sir Peter MacCallum Department of Oncology, The University of Melbourne, Victoria, 3010 Australia

**Keywords:** Cancer clinical trials, Communication, Patient experience, Mixed-method

## Abstract

**Background:**

As cancer therapies increase in their complexity, effective communication among patients, physicians, and research staff is critical for optimal clinical trial management. Currently, we understand little about on-trial communication practices and patient trial experiences over time. This mixed-method study explored patient experiences of participating in a clinical drug trial at different time points, focussing on patient communication with trial staff.

**Methods:**

Patients enrolled in clinical drug trials conducted at the Parkville Cancer Clinical Trials Unit were invited to complete a tailored online survey and/or a qualitative interview. Patients were recruited to three cohorts based on time since the first trial treatment: new (≥ 1 to ≤ 13 weeks), mid- (≥ 14 to ≤ 26 weeks), and long-term (≥ 52 weeks) trial patients. Descriptive statistics were calculated for survey responses. Interview data were analysed thematically with a team-based approach. Survey and interview data were integrated at the intepretation stage.

**Results:**

From May to June 2021, 210 patients completed a survey (response rate 64%, 60% male), 20 completed interviews (60% male), and 18 completed both. More long-term trial patients (46%) participated than new (29%) and mid-trial patients (26%). Survey data showed high (> 90%) patient satisfaction with the provision of trial information and communication with trial staff across trial stages, and many reported trial experiences as *above and beyond* standard care. Interview data indicated that written trial information could be overwhelming, and verbal communication with the staff and physicians was highly valued, especially for enrolment and side effect management among long-term patients. Patients described the key points along the clinical trial trajectory that merit close attention: clear and well-communicated randomisation practices, reliable pathways for side effect reporting and prompt response from the trial staff, and end-of-trial transition management to avoid a sense of abandonment.

**Conclusion:**

Patients reported high overall satisfaction with trial management but outlined key pinch points requiring improved communication practices. Establishing a range of effective communication practices among trial staff and physicians with patients in cancer clinical trials may have a wide range of positive effects on patient accrual, retention, and satisfaction.

## Introduction

Clinical trials are a crucial component of evidence-based cancer medicine as they facilitate the development of novel cancer therapies that improve patient outcomes. For many cancer patients, clinical trials offer the best treatment available, especially for those with advanced or rare cancer where conventional treatment options may have been exhausted or are unavailable. The experimental nature of cancer clinical trials, however, means the potential risks and benefits to patients are uncertain, bringing complexity to decisions about enrolling and staying on trial. Critical to managing this inherent uncertainty is effective communication among patients, physicians, research staff, and family [1–4].

Clinical trial research staff in particular play a vital role in ensuring that trial information is clearly and effectively communicated to patients over the course of a trial [5]. At enrolment, the trial staff are responsible for providing adequate trial information to promote patient understanding of clinical trial requirements and facilitate informed consent. It is well documented that decision-making about participating in cancer clinical trials is a complex process with an overwhelming volume of both written and verbal information, often provided at times of vulnerability for patients and their families [6–9]. Some patients misunderstand the purpose and implications of their clinical trial, particularly in relation to how it may benefit them, and the risks of their involvement, raising concerns about informed consent [10–15].

While there is a wealth of research on recruitment to cancer clinical trials, we understand little about on-trial communication practices and patient experiences over time. Ongoing communication throughout a trial is essential to ensure patients’ potential side effects, health outcomes, and expectations are well managed. Quality communication between patients and their trial team is a good predictor of trial retention [5, 16]. Communication practices and approaches in cancer clinical trials, however, are highly context-dependent. Effective methods in one setting may not be in others, making service evaluation an important aspect of contemporary clinical trial management.

As cancer therapies increase in their complexity [17], so do the challenges for patients [18, 19]. Trial drugs have a growing range of unique toxicities that require specific management (e.g. immunotherapy), have sometimes intricate randomisation protocols (e.g. for combinational therapy), and can require patients to remain on trial for extended periods, potentially for life [17, 20]. Communication between trial patients, staff, and physicians must constantly adapt to these new therapies; however, patient experiences of being on trial for long periods remain understudied. Understanding patient experiences will assist with developing safer and better quality care and greater patient satisfaction. Improved long-term trial engagement would in turn yield a higher quality of evidence from clinical trials and a cost-effective return from significant investment.

This study was nested within a larger service evaluation at a specialist clinical trials unit in a public cancer hospital in Melbourne, Australia: the Parkville Cancer Clinical Trials Unit (PCCTU). This study aimed to explore patient experiences of participating in a clinical drug trial from beginning to end, focussing on trial enrolment, pre-screening, care plans, and first treatment; relevance and usefulness of trial information; quality and accessibility of communication with clinical trial teams over time, including adverse events; and long-term trial participation and communication experiences.

## Materials and methods

This study employed a two-phase, concurrent mixed-method approach to explore patient experiences of clinical drug trial participation at the PCCTU, Peter MacCallum Cancer Centre (Peter Mac). Phase 1 consisted of cross-sectional online surveys, which were supplemented by concurrent phase 2 qualitative interviews with a subset of purposively sampled participants. A mixed-method approach was taken to gain and compare both group-level and individual-level trial experiences and develop a more complete understanding of trial participation [21]. Data were collected from April to June 2021, analysed in parallel and integrated at the interpretation stage [22]. This study was approved by the Peter MacCallum Cancer Centre Human Research Ethics Committee (HREC LNR/60091/PMCC).

### Participants

#### Population and sampling

As the largest clinical trials service in Australia, the PCCTU provides care to a highly heterogeneous patient population across the full cancer spectrum (i.e. breast, gynaecological, haematological, melanoma, general medical oncology). Consequently, patients at the PCCTU are enrolled in a wide range of cancer drug trials, varying by drug administration (e.g. tablet and infusion), drug type (e.g. immunotherapy, chemotherapy, combination therapy), and clinical trial phase (i.e. 1–3). Consistent with our aims of understanding the overall clinical trial experience—agnostic to trial, drug, and cancer type—at different time points, the sampling frame consisted of all consecutive patients enrolled in clinical drug trials administered through the PCCTU with an anchor date of April 2021. Patients were eligible for inclusion if they read, understood, and spoke English, and if they met one of the cohort criteria below:

- Cohort 1, newly enrolled patients: ≥ 1 to ≤ 13 weeks post-first treatment
- Cohort 2, mid-trial patients: ≥ 14 to ≤ 26 weeks post-first treatment
- Cohort 3, long-term trial patients: ≥ 52 weeks post-first treatment

Patients enrolled in treatment-naïve acute leukaemia studies were excluded due to the extensive inpatient stay involved in their care.

The time criteria for each cohort were designed a priori in collaboration with PCCTU staff to reflect general patterns in trial activity and intensity. Cohort 1 criteria were designed to capture patient experiences of learning about a trial, enrolment, and commencement, which is an intense period for patients that invariably occurs within the first 3 months of a trial. Cohort 2 criteria were designed to investigate ongoing trial experiences, specifically the identification and management of side effects, and to capture the acclimation process for patients as they get used to the logistics and nature of being on trial after the intensive onboarding process. Cohort 3 criteria were designed to capture the experiences of longer-term trial patients, potentially on trials with extensive follow-up or being maintained on trial drugs long term for clinical reasons.

For qualitative interviews, purposive sampling was employed to ensure an approximately equal variation of key demographic and health characteristics based on gender, age, education, PCCTU treatment team, time spent on trial, and experiences of side effects from trial drugs [23].

#### Recruitment

A list of all potentially eligible patients was extracted from PCCTU clinical trial records. The research team cross-checked patients with electronic medical records to confirm eligibility before inviting them to the study via email with a survey link and research team contact details. The survey landing page contained study and consent information. Completion of the survey indicated participant consent. All responses were anonymous to ensure confidentiality. At the end of each survey, participants were invited to provide contact information to complete a qualitative interview or could telephone the research team directly. Participants who opted for an interview were assessed against the purposive sampling criteria, and those eligible were contacted to schedule an interview.

### Data collection and analysis

#### Surveys

Two purpose-built, descriptive surveys were developed in collaboration with PCCTU leadership and were based on key evaluation aims of the service. Quantitative and open-text data were collected cross-sectionally (Supplement 1). The first survey addressed new trial participant experiences (cohort 1), comprising 35 items across four domains: (1) demographics; (2) trial information and consent; (3) screening, care plans, and first treatment; and (4) clinical trial team communication. The second survey was for mid- and long-term trial participants (cohorts 2 and 3), comprising 39 items across four domains: (1) demographics, (2) communicating and managing side effects, (3) managing ongoing treatment, and (4) clinical trial team communication. Both surveys were administered online and managed using Research Electronic Data Capture (REDCap) tools hosted at Peter Mac [24]. Descriptive statistics were calculated to summarise survey responses. Open-text responses from the survey were extracted to Excel and analysed using qualitative content analysis [25]. AB performed a within-question content analysis per open-text question, organising responses into inductively developed content categories before RFS and AH performed secondary checking. The number of responses was reported for each content category.

Where possible, and to assist with integrating survey and qualitative interview data, RFS then conducted a cross-question content analysis to link issues shared by each cohort: content categories were grouped based on shared meaning or content across open-text questions (e.g. issues with side effects). AB, RFS, and AH later met to compare these broad content categories with the codebook and thematic domain summaries from qualitative interview analysis to inform the overall interpretation of our findings. Data integration occurred at the interpretation and reporting stage; illustrative open-text responses from surveys were used to add a group-level element to our individual-level interpretations of qualitative interviews.

#### Qualitative interviews

Interviews were conducted over the phone or in person at Peter Mac, depending on participant preferences. Interviewees provided verbal consent prior to beginning their interview. A semi-structured interview guide was used to facilitate each interview, focusing on participants’ trial experiences, communication with the clinical trial team and their support needs (Supplement 2). Interviews were audio-recorded and transcribed verbatim by a professional transcription service. AB (qualitative researcher in training) and RFS (qualitative expert) conducted all the interviews.

All transcript data were analysed using team-based, codebook thematic analysis [26, 27]. AB, RFS, and AH read all transcripts for data familiarisation. A preliminary codebook was then developed, listing possible codes, their definitions, and an example. AB then coded the first 10 transcripts using the preliminary codebook; RFS reviewed all coding, iteratively adjusting and updating the codebook. This process was repeated using the updated codebook to code the remaining transcripts. RFS then developed domain summaries for each code and their associated transcript data before the final themes were generated based on team consensus. QSR International NVivo™ (2020) was used for qualitative data management.

## Results

**Fig. 1:**
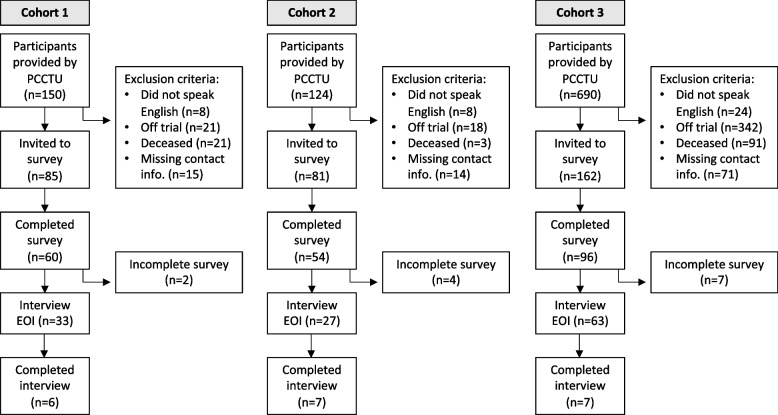
Flowchart of EXPECT study participation by cohort

After the assessment against the study eligibility criteria, 327 individuals were invited to participate, and 210 completed the survey (response rate 64.2%) (Fig. 1). Of the 210 survey respondents, 123 (58.6%) expressed interest in completing a qualitative interview. In total, AB and RFS interviewed 20 participants from May to June 2021. Interviews took an average of 30.45 min (range 16–44 min); 18 were completed over the phone and two in person at Peter Mac. Two participants opted to complete an interview only.

### Participant characteristics

The survey and interview samples were similar. There were more male participants (60%), and the mean age was approximately 60 years with a similar variance (Table 1). Most participants in each sample were trial-naïve (78.6% of survey vs. 65.0% of interviewees). There was an even spread of interviewees across each cohort: cohort 1 (*n* = 6, 30%), cohort 2 (*n* = 7, 35%), and cohort 3 (*n* = 7, 35%).

**Table 1:** Survey and interview participant characteristics

Demographic	Survey total (*n* = 210)^a^	Survey cohort 1 (*n* = 60)	Survey cohort 2 (*n* = 54)	Survey cohort 3 (*n* = 96)	Interview sample (*n* = 20)^b^
**Age (years)**					
Mean (range)	61.8 (21–85)	60.9 (35–82)	61.4 (21–85)	62.7 (29–82)	60.6 (25–82)
**Gender,** *** n*** ** (%)**					
Male	126 (60.0)	35 (58.3)	34 (63.0)	57 (59.4)	12 (60.0)
Female	83 (39.5)	24 (40.0)	20 (37.0)	39 (40.6)	8 (40.0)
**Previous cancer clinical trial experience**					
Trial naïve	165 (78.6)	49 (81.7)	44 (81.5)	72 (75.0)	13 (65.0)
On trial before	44 (21.0)	10 (16.7)	10 (18.5)	24 (25.0)	5 (25.0)
**Metastatic cancer**					
Yes	114 (54.3)	42 (70.0)	23 (42.6)	49 (51.0)	7 (35.0)
No	79 (37.6)	13 (21.7)	26 (48.1)	40 (41.7)	10 (50.0)
Unsure	17 (8.1)	5 (8.3)	5 (9.3)	7 (7.3)	1 (5.0)
**Long-term trial participation**					
On active treatment	–	–	–	60 (62.5)	–
Under long-term observation	–	–	–	36 (37.5)	–
**Highest attained level of education**					
Non-tertiary	121 (57.6)	34 (56.7)	34 (63.0)	53 (55.2)	7 (35.0)
Tertiary	88 (41.9)	25 (41.7)	20 (37.0)	43 (44.8)	11 (55.0)
**First language**					
English	198 (94.3)	59 (98.3)	48 (88.9)	91 (94.8)	20 (100)
Others	11 (5.2)	0	6 (11.1)	5 (5.2)	0 (0)
**Current relationship status**					
Single	21 (10.0)	6 (10.0)	5 (9.3)	10 (10.4)	2 (10.0)
Married/de facto/partnered	165 (78.6)	46 (76.6)	42 (77.8)	77 (80.2))	14 (70.0)
Separated/divorced/widowed	23 (11)	7 (11.7)	7 (13.0)	9 (9.4)	2 (10.0)
**Current employment**					
Employed	91 (43.3)	26 (43.3)	26 (48.1)	39 (40.6)	8 (40.0)
Not employed	20 (9.5)	9 (15.0)	6 (11.1)	5 (5.52)	2 (10.0)
Retired	97 (46.2)	24 (40.0)	22 (40.7)	51 (53.1)	8 (40.0)

^a^Missing data *n* = 1 from survey cohort 1 and *n* = 2 from qualitative sample

^b^Interview sample is separate, but mostly drawn from the survey sample (bar *n* = 2). Counts from this column do not contribute to the survey totals (*n* = 210)

### Integrated analysis

Our inductive thematic analysis produced several interrelated themes describing how participants experienced being on a clinical trial at the PCCTU and the different ways they communicated with the trial staff. Survey and qualitative data are integrated and organised into themes according to participant trial stage: Stage 1: going on trial; Stage 2: being on trial; and Stage 3: coming off the trial. Each theme is supported by de-identified participant quotes. Participant descriptors (gender, age range, and cohort) are provided in parentheses to contextualise responses.

### Stage 1: Going onto a clinical drug trial

Theme 1 explored the varying processes and experiences of going onto a clinical drug trial across participants. Sub-themes identified included the variety of pathways to trial enrolment, the range of information available to support trial enrolment decision-making, and patients' ability to navigate trial eligibility and randomisation processes.

#### Pathways to trial

Participants mostly elected to enrol in a clinical trial by recommendation from their oncologist, although some reported seeking out specific trials themselves using a variety of sources (e.g. media or family connections) and requesting their oncologist to arrange it. Once on trial, there were mixed reports about keeping participants’ treating doctor or general practitioner (GP) *in the loop* with patient trial progress (F, 70–79, C3). Reports suggested that GPs being looped in from trial start provided a safety net for dealing with non-trial, health-related issues locally.

#### Decision-making and understanding of study information

Nearly all cohort 1 participants (*n* = 60) reported that the written (95%) and verbal trial information (96.7%) was easy to understand, that they felt confident in their understanding of what they had consented to (91.7%), and had sufficient time to ask questions when they enrolled (93.3%) (Table 2). Study information was predominantly provided via patient information and consent forms (PICFs), either in print or email, and supplemented with at least one conversation with the trial team prior to enrolment. Having both comprehensive verbal and written information was valued by participants, especially in managing expectations:

[The PICF] was quite good in that it wasn’t offering any false hopes or promises, it was quite specific saying [that] it may have no difference whatsoever, as if you weren’t on the trial. (M, 60–69, C2).

Once I started reading [the PICF] I realised it was written in plain speak and it addressed most of the questions I had anyway, but they were always available to answer any questions and go over anything. (F, 20–29, C1).

**Table 2:** Cohort 1 experiences of communication about clinical trial information, consent, trial eligibility, and first treatment (*n* = 60)

	*n*	%
**Was the written trial information easy to understand?**		
Yes	57	95.0
No	2	3.3
**Was the verbal/spoken trial information easy to understand?**		
Yes	58	96.7
No	2	3.3
**Did you have enough time to ask questions about the trial information?**		
Yes	56	93.3
No	3	5
**Did you find any parts of the trial information confusing?**		
Yes	3	5.0
No	53	88.3
Unsure	4	6.7
**Are you confident describing the function of the trial drug?**		
Yes	52	86.7
No	4	6.7
Unsure	4	6.7
**When consenting, did you feel confident you understood what you were signing up for?**		
Yes	55	91.7
No	2	3.3
Unsure	3	5.0
**Was there any information that was missing or would have been useful to know when consenting?**		
Yes	11	18.3
No	43	71.7
Unsure	5	8.3
**Were you given information about your eligibility before your first treatment?**		
Yes	52	86.7
No	3	5.0
Unsure	4	6.7
**Did you understand the information about your trial eligibility?**		
Yes	51	85.0
No	2	3.3
Unsure	6	10.0
**Were you given information about your treatment schedule before starting?**		
Yes	58	96.7
No	1	1.7
**Did you feel well prepared for your first infusion or treatment?**		
Yes	57	95
No	1	1.7
Unsure	1	1.7

Missing data *n* ≤ 1

PICFs were a primary source of reference material for participants and their families; however, verbal conversations were treated by many as the real *run down* (M, 70–79, C3) as they were an opportunity to ask questions and gauge the opinions of the trial team and/or treating oncologist about the trial. These conversations held particular importance because, despite positive responses about written information in the survey, a common sentiment from qualitative data was that PICFs were too long and detailed, making study information difficult to digest in the first instance: *The size of the [PICF] overwhelmed me initially* (F, 20–29, C1).

[PICFs] go into too much detail, detail that people don’t need to know. The information [the trial team] give should be what [patients] need to know, not what you think an expert would know. (M, 70–79, C2).

Receiving *too much information* (F, 20–29, C1), both written and verbal, was common at screening and consent. As a result, participants placed a great deal of trust in their doctors’ endorsement of the trial. Some described signing their PICF as a formality, rather than trying to read and understand the information within.

[My consent] had to be written and signed off as well as verbal, I mean there was a lot of documents, a lot of information. I was okay [with it], I’m not an avid reader and I sort of trust these people and the verbal [explanation] was enough for me [to consent]. (M, 70–79, C3).

I had to sign the consent form and I went through pages of just… it was pretty much over my head with words and things like that. We just read it and I signed it and that’s it. (M, 40–49, C3).

Importantly, trying to understand complex study information with metastatic cancer or soon after a cancer diagnosis was difficult. One participant described that being *floored by [their] diagnosis* meant important trial information went *over [their] head* (M, 60–69, C2) during the trial consent process. Another metastatic participant described that a sense of desperation permeated their enrolment.

I remember going in there and there were sheets, forms to sign. I honestly couldn’t tell you what went backwards and forwards. When you’re in that condition, and look, I was at a stage where I wasn’t far from dead, I could hardly breathe, I could hardly walk. The cancer had basically gone into the blood, so I really wasn’t thinking straight anyway and when you’re in that mood and that sort of condition, all you want is somebody who’s going to help you. (M, 70–79, C2).

Moreover, 11 cohort 1 participants (18.3%) felt there was missing information when they consented to the trial (Table 2). In the open text, they reported wanting to know more about the options to change drug dosage, the risk of side effects compared to other trial participants, the differences between side effects and cancer symptoms, and information about trial scheduling.

#### Eligibility and randomisation

Eight participants (13%) of cohort 1 did not understand or were unsure about their trial eligibility (Table 2). From 50 open-text responses, cohort 1 participants indicated that eligibility information was mainly provided verbally (*n* = 40, 80%) and only sometimes in combination with written information (*n* = 8, 16%). Two participants reported that randomisation processes made eligibility information difficult to understand, with implications for setting realistic expectations, organising travel, and childcare as different trial arms had different requirements.

It is a randomised trial, but I wasn’t told which group I had been assigned to until I realised that, had I been in the other group, I would have been receiving an additional treatment. (M, 80-89, C1).

I think the randomisation should have happened before eligibility for mental state plus organising lifts, childcare etc. well ahead of time… I’m a single parent so I don’t have like a backup person to have money coming in. (F, 40–49, C1).

Information about randomisation was sometimes poorly communicated. One participant felt she was misled and given a *false sort of idea* of being in the treatment arm when preparing for appointments (F, 40–49, C1). She was understandably disappointed when she was randomised differently:

I found it quite distressing when I didn’t get into… what I perceived as the better group… I found that quite disappointing and a little bit traumatic. (F, 40–49, C1).

### Stage 2: Being on a clinical drug trial

This theme addresses three specific aspects of being on trial: on-trial communication, experiences related to side effects, and trial care as an improvement on usual care.

#### On trial communication

The majority of cohort 2 and 3 participants (*n* = 150) reported contacting the clinical trial team at least once with queries or questions (74.7%) (Table 3). Common queries included supplementary medication advice, medical test results, appointment scheduling, and side effect and symptom management. While on trial, participants reported their trial team was good at managing/changing appointments (88.7%) and helping them understand the reason for their different appointments (98.7%) (Table 3). Common negative experiences reported in open text by 20 participants (13.3%) included the turnover of trial staff, not feeling heard by the trial staff, slow response times to queries, and mistiming treatment schedules. Notably, some long-term trial participants found it *tedious* to rebuild working relationships with new staff as they change over time:

[After staff changeover] I went back to being invisible and having to sort of justify being there a bit, they didn’t have the history, you had to go back to the beginning and explain things – so it got a bit tedious after the fourth time. (F, 70–79, C3).

**Table 3:** Cohort 2 and 3 experiences of communication about clinical trial treatment management (*n* = 150)

	Total (*n* = 150)		Cohort 2 (*n* = 54)		Cohort 3 (*n* = 96)	
	*n*	%	*n*	%	*n*	%
**Have you ever needed to contact the clinical trial team with questions?**						
Yes	112	74.7	38	70.4	74	77.1
No	36	24.0	16	29.6	20	20.8
Missing	2	1.3	–	–	2	2.1
**Have you ever had trouble getting in contact with the clinical trial team?**						
Yes	17	11.3	4	7.4	13	13.5
No	131	87.3	50	92.6	81	84.4
Missing	2	1.3	–		2	2.1
**Was the clinical trial team able to answer questions about accommodation/travel reimbursement?**						
Yes	79	52.7	31	57.4	48	50.0
No	7	4.7	1	1.9	6	6.2
Not applicable to me	37	24.7	14	25.9	23	24.0
Missing	27	18.0	8	14.8	19	19.8
**Was the clinical trial team good at helping you manage your other health conditions?**						
Yes	103	68.7	37	68.5	66	68.8
No	7	4.7	2	3.7	5	5.2
Not applicable to me	37	24.7	14	25.9	23	23.9
Missing	3	2.0	1	1.9	2	2.1
**Was the clinical trial team good at helping to arrange contact with supportive care services?**						
Yes	53	35.3	23	42.6	30	31.3
No	3	2.0	0	0.0	3	3.1
Not applicable to me	92	61.3	31	57.4	61	63.5
Missing	2	1.3	–	–	2	2.1
**Was the clinical trial team good at helping you manage or change your appointments?**						
Yes	133	88.7	48	88.9	85	88.5
No	2	1.3	0	0.0	2	2.1
Not applicable to me	13	8.7	6	11.1	7	7.3
Missing	2	1.3	–	–	2	2.1
**Was the clinical trial team good at helping you understand what your different appointments were for?**						
Yes	148	98.7	54	100.0	94	97.9
No	0	0.0	0	0.0	0	0.0
Missing	2	1.3	–	–	2	2.1
**Have you had any experiences where communication with the clinical trial team left you feeling unhappy or that your query had not been resolved?**						
Yes	20	13.3	4	7.4	16	16.7
No	128	85.3	50	92.6	78	81.2
Missing	2	1.3	–	–	2	2.1

#### Treatment-related side effects

##### Expectations about side effects

Nearly a quarter of cohort 2 and 3 participants (*n* = 36/150, 24%) reported being admitted to the hospital due to side effects at least once (Table 4). Reported side effects were consistent with what was presented in the trial information; no side effects were described as unexpected or shocking. Side effects, whether trial-related or from previous conventional treatment, were seen as routine and something patients had to *put up with* (M, 60–69, C2) and got *better at managing* over time (F, 20–29, C1). Many described expecting much worse from trial drugs than they had already experienced from conventional treatment (e.g. chemo- and radiotherapy):

I expected worse because the side effects of [my previous] chemo and radiation were so bad. So I thought well, logically, if you’re, if immunotherapy is activating your immune system, I could have all sorts of things happening. I’m prepared for the worst, I was prepared for rashes and feeling a lot tireder and feeling a lot sicker, and that hasn’t happened. (F, 60–69, C2).

**Table 4:** Cohort 2 and 3 experiences of communication about side effects (*n* = 150)

	Total (*n* = 150)		Cohort 2 (*n* = 54)		Cohort 3 (*n* = 96)	
	*n*	%	*n*	%	*n*	%
**Did you find the written information about side effects easy to understand?**						
Yes	140	93.3	51	94.4	89	92.7
No	9	6.0	3	5.6	6	6.3
**Did you find the verbal/spoken information about side effects easy to understand?**						
Yes	142	94.7	52	96.3	90	93.8
No	6	4.0	2	3.7	4	4.2
**Did you find the information about side effects useful?**						
Yes	141	94.0	51	94.4	90	93.8
No	7	4.7	3	5.6	4	4.2
**Was there anything missing in the information about side effects?**						
Yes	22	14.7	8	14.8	14	14.6
No	125	83.3	46	85.2	79	82.3
**Was it easy to get your questions about side effect answered by your clinical trial team?**						
Yes	135	90.0	49	90.7	86	89.6
No	5	3.3	3	5.6	2	2.1
Unsure	8	5.3	2	3.7	6	6.3
**How was the information about side effects provided to you?**						
Patient information and consent form	130	86.7	48	88.9	82	85.4
Study doctor	110	73.3	38	70.4	72	75.0
Study nurse/coordinator	99	66.0	37	68.5	62	64.6
Nurse giving treatment	56	37.3	18	33.3	38	39.6
Pharmacy	27	18.0	13	24.1	14	14.6
Internet	25	16.7	12	22.2	13	13.5
Drug company info	25	16.7	8	14.8	17	17.7
Others	3	2.0	0	0.0	3	3.1
**Have you ever been to hospital due to a side effect?**						
Yes	36	24.0	13	24.1	23	24.0
No	112	74.7	41	75.9	71	74.0

Missing data *n* ≤ 2

Participants shared that possible side effects were listed in written study documents, which they used to ask questions about side effects of concern. PICFs were the most common source of information about side effects (86.7%), followed by study doctors (73.3%) and study/nurse coordinators (66.0%) (Table 4). The 22 participants (14.7%) who reported missing information about side effects explained wanting more information about pain, severity of side effects, effects of trial drugs on vaccines (for COVID), impacts on sleep, long-term drug effects (e.g. muscle wastage), visual disturbances, and managing constipation (Table 4).

Some participants felt that focussing on potential side effects in detail could prompt hypervigilance about bodily symptoms: *If something happens you immediately think, ‘Oh my God, is that a side effect? What’s happening?’ So, there’s a very fine line I think between being well-informed and too informed* (F, 50–59, C2). In contrast, one metastatic participant shared that potential side effects were a non-issue to him as his prognosis was too poor to decline even potentially risky experimental treatment.

Some of the sheets that I’ve been given, you know, [saying] ‘this [drug] may cause harm, and it may do this…’ I really don’t care. Without it I’m going to die anyway. If you’re going to say to me, ‘If you take this medicine, it might hurt you but it will hurt me hell of a lot more if I don’t take it…’. Look, you’d be completely stupid if you sat down and read it and thought, ‘Oh, if I take this medicine my big toe might drop off’, now hang on, without it you’ll die of cancer. You’ve got to be sensible about it. (M, 70–79, C2).

Participants in phase 1 trials reported receiving limited information about side effects at enrolment but understood that this was because data were not yet available, and they nonetheless felt closely monitored.

##### Trial team support in managing side effects

Contacting the trial team about side effects was generally reported positively. Being encouraged to take advantage of the available and effective medications to manage side effects gave patients confidence about speaking to the trial team and having their needs met. Some reported getting tips from the trial team about managing side effects, and others were encouraged also to use their GP where possible to manage side effects locally. A minority (9%, *n* = 13), however, found it difficult or were unsure how to get answers from the trial team about side effects. Although rare, some reported trial staff being dismissive to their questions, especially about minor side effects:

I have several side effects some mild and some severe and I got the impression that they thought I was making some up. (M, 70-79, C2)

In terms of managing more severe side effects, participants reported receiving a range of different support from the trial team. One positive was that being encouraged by the trial team to present at the emergency department, even if for a fever, was validating of patients’ high risk while on trial.

It’s sort of a weird scenario because like normally if you don’t have a chronic illness you wouldn’t go to the hospital for a fever, so it was sort of getting that reassurance that you were doing the right thing to go to hospital to do that because I haven’t had to do that before. (F, 40–49, C1).

One challenging aspect was contacting the trial team with side effects outside of business hours. The inability to get tailored advice about side effects from generic hospital services out-of-hours meant that some participants waited to seek help about their side effects until they knew their trial team would be available. For other participants, it was difficult to gauge the importance or urgency of their side effects, and they delayed contacting the trial team to avoid wasting the staff’s time with potentially minor concerns:

[My fever] did rise to about 38.5 a couple of times but I sort of held off [contacting the trial team] because I thought I don’t want to waste their time because I know that this is a side effect… (M, 70–79, C3).

Once admitted to the hospital due to side effects, two participants described that staying in contact with the trial team was challenging, leaving them to advocate for their trial-specific needs without support from their trial team as inpatients.

#### Trial care as an improvement on usual care

Finally, participants overwhelmingly reported positive experiences while on trial and that being on trial was a drastic improvement to standard care. Many felt well *looked after* (F, 70–79, C3) and *of interest to someone* (F, 50–59, C2), variously describing their trial care as *above and beyond* (M, 30–39, C1) and highly organised compared to usual care.

Before I started on the drug trial, the doctor was kind… but no one was interested in me and my experience wasn’t relevant. While you’re on the trial you get a lot more attention, people want to know how you’re going and you do get a bit of extra TLC, which is probably very egocentric but everyone on the trial experiences it and I think that is therapeutic in a way. (F, 70–79, C3)

### Stage 3: Coming off a clinical drug trial

This theme described the experiences of a considerable proportion of participants in both samples (21% and 25%, respectively) that had previously transitioned off a clinical drug trial (Table 1). In particular, participants reflected on the negative or challenging aspects of transitioning back to usual care and the worries and concerns which accompanied a trial drug not being successful in treating their cancer.

#### Transition back to standard care

Coming off trial was described as a significant change. Transitions from highly attentive on-trial care to standard care that *leaves a lot to be desired* (M, 70–79, C3) were difficult for some participants.

I suppose you tend to get rather spoiled by your trial nurse because, as I said, nothing was too much trouble, whereas being off the trial, well, yeah, that leaves a lot to be desired… like being part of the general population again… it makes life a bit more difficult. (M, 70–79, C3).

For other participants, the possibility that they could go off the trial, and the conditions and decision-making to take them off, was poorly communicated. This meant it *was a bit of a shock* when they were transitioned off trial with little notice (M, 30–39, C1).

They probably mentioned it in all the documents they’ve given me but there’s a lot to read and you don’t always retain everything either, like, I’m not in the medical profession. But maybe if they could just say, ‘If things go this way you won’t continue on the trial’… it would have been good maybe if they could just explain that a bit more that you’ll be on the trial until something like this happens and then you’ll be off. (M, 30–39, C1).

One facet of coming off trial was a sense of worry about *wasting time* while being on an experimental drug that proved to be ineffective (M, 30–39, C1). One participant reflected: … *people could feel used especially if the drug doesn’t work* (F, 70–79, C3). This feature came across strongly for one participant who went directly from diagnosis to a clinical trial as their first treatment option. He was concerned about whether going on trial was a potentially poor decision in hindsight when conventional treatment (e.g. surgery) was available.

Now that I’m off [the trial] we’ve got concerns of, ‘Did I waste time?’, you know, ‘Did I do the wrong thing for me personally?’ So, I guess that’s something that’s not really clear to me either. (M, 30–39, C1).

## Discussion

This study examined the communication and experiences of cancer patients at different stages of a clinical drug trial and makes an important contribution to understanding trial experiences beyond decision-making to enrol and the informed consent process. Our mixed-method approach identified overall patient satisfaction with trial management, especially the provision of trial information, and high levels of communication between patients and trial staff over time. Concurrent in-depth interviews identified key points along the clinical trial trajectory that merit further attention, namely randomisation practices, side effect reporting and response from the trial staff, and end-of-trial transition management.

### Views on written and verbal information

There is currently a limited consensus on the most effective way of communicating trial information to participants. Written and verbal styles of communication are reportedly effective, and while some patients find trial information overwhelming, others report wanting more detailed information, especially about study drugs and procedures [10, 15, 28, 29]. Patients in the current study also had a range of preferences on the length, complexity, and mode of trial information, indicating that ‘one-size-fits-all’ approaches are unlikely to meet the varying needs of all patients. Introducing more personalised trial information provision in flexible, accessible, and responsive formats could improve patient understanding at trial enrolment [30].

A substantial minority of newly enrolled trial patients (18%) wanted more information about drug dosage, relative risk of side effects, and trial eligibility, suggesting drug-specific information may be deserving of special attention at recruitment. Written information, mostly provided as PICFs, was a valuable reference material for patients while on trial, but less useful when needing guidance in emergency situations (i.e. when experiencing severe side effects). The sizeable rate of side effect-related hospital admissions (24%) among mid- and long-term trial patients suggests that providing tailored side effect information and resources for what to do when patients experience them could be useful.

In agreement with other studies, we found that patients’ decisions to enrol in a clinical cancer trial were mostly influenced by emotions and trusting relationships with trial staff rather than engaging with often dense written information [7, 10, 15]. Some patients who found PICFs difficult to read viewed them as a legal formality and preferred learning about study information through verbal conversations with their trusted treating team. Understanding the key communication strategies of oncologists and trial staff in clinical trial settings stands out as an important area of future research that could improve patient-centred care and trial understanding [31].

Moreover, departing from heavily written information-based consent processes may better align with the varied cognitive, affective, and social processes that patients employ in deciding to enrol in clinical trials [15], especially those in vulnerable positions of existential challenge [9, 32]. Our study reflected similar findings, where patients at end of life or who had limited treatment options described choosing to enrol in the trial as they were desperate for care. Implementing effective interventions such as enhanced consent forms and extended discussions could improve participant understanding of clinical trials at enrolment in these contexts [33]. However, how these more time-intensive approaches may integrate into the operations of increasingly busy clinical trials units that are subject to resourcing constraints and trial sponsor recruitment targets remains unclear.

### Randomisation

The way in which randomisation is explained can be a barrier to trial enrolment, especially among low literacy populations, given its complex and technical nature [34]. We found that patient misunderstandings about randomisation had implications for setting realistic expectations about trial outcomes and planning for required time commitments per trial arm. Potential strategies to improve understanding about randomisation could include mock randomisation scenarios, with plain language summaries of the time requirement per trial arm, so patients can fully consider enrolment considering caring and/or work responsibilities. Other studies suggest linguistic strategies such as the use of culturally grounded metaphors (e.g. the probability of the sex of a baby) instead of standard lay metaphors that appeal to notions of gambling (e.g. roll of a die) could improve understanding of randomisation [35, 36]. Such strategies merit further investigation as trials become more complex and more attention is paid to increasing trial recruitment from underserved and culturally diverse communities.

### Being on trial

A novel aspect of our study was that we captured a large number of patient views about being on trial long term. Communication with the trial team during the mid-trial period (≥ 14 weeks to ≤ 26 week post-first treatment) was critical, especially when managing side effects. Though rare, prompt after-hours response from the trial staff when patients had side effect emergencies was reportedly poor. To help ensure patients are well-managed while on trial for extended periods, online self-report monitoring systems of adverse events and side effect bother with clear pathways to clinical follow-up could be employed [37, 38]. Furthermore, ‘long haul’ trial participants (≥ 52 weeks from first treatment) reported being highly acclimated and familiar with the healthcare system and with treatment and trial procedures. Indeed, some shared having to bring new staff up to speed. A buddy system that pairs new and experienced trial patients could help settle new patients and help trial management overall.

### Transitions off trial

Communication at clinical trial exit is also an important, but oft-neglected aspect of clinical trial management [39]. Some patients in our sample described being unfamiliar with the reasons they were withdrawn from a trial. Because trial eligibility was mostly communicated verbally, this could be due to patients having limited materials (e.g. written or audio-recorded summary) to refer to. Furthermore, patients transitioning off trial have reported feeling a sense of abandonment and distress when reasons for withdrawal and post-trial processes are poorly communicated [39, 40]. This was also true for a small number of patients in our study, and, similar to other reports, a notable aspect of being transitioned off trial for one participant was the feeling of ‘wasting time’ when the trial drug failed [40, 41]. Developing materials to assist patients in understanding the withdrawal criteria and readying alternative treatment or palliative options when transitioning patients off trial may assist in making them feel more in control at this time. In combination, these findings point to the need for more targeted research on how best to transition patients off the trial in ways that respect and honour their contribution to the research.

### Strengths and limitations

Our mixed-method design provided both the general population and individual patient views of trial participation across the trial trajectory. The participation rate was high (64.2%), especially among males, who are poorly represented in psychosocial oncology research [42]. We were unable to report on comparisons between invited participants and non-participants due to our ethics approval, potentially obscuring any selection bias in our sample. There was a good spread of participants at each trial time point, especially long-term trial participants, whose perspectives are generally lacking from the literature. The study was conducted at a well-resourced tertiary cancer centre that specialises in research with a non-random sample, limiting generalisability. Critically, due to taking an in situ service evaluation approach, we did not specifically sample for culturally and linguistically diverse participants or for low health literacy, nor did we aim to stratify results by advanced cancer stage or trial type, which are known factors that affect clinical trial experiences and informed consent [8, 43]. Improving precision when reporting patient communication experiences, by including additional pertinent socio-demographic and trial-related variables, is an important avenue for future research.

## Conclusion

This study confirmed the importance of timely, patient-centred, honest, and easy-to-navigate communication between cancer clinical trial participants and staff. We conclude that establishing a variety of effective communication practices among trial staff and physicians with patients in cancer clinical trials may have a wide range of positive effects on patient accrual, retention, and satisfaction that warrant further enquiry.

## Data Availability

The datasets generated and/or analysed during the current study are not publicly available to ensure the anonymity of participants but may be available in part from the corresponding author upon reasonable request.
